# Disorder of G2-M Checkpoint Control in Aniline-Induced Cell Proliferation in Rat Spleen

**DOI:** 10.1371/journal.pone.0131457

**Published:** 2015-07-20

**Authors:** Jianling Wang, Gangduo Wang, M. Firoze Khan

**Affiliations:** Department of Pathology, University of Texas Medical Branch, Galveston, TX 77555, United States of America; University of Central Florida, UNITED STATES

## Abstract

Aniline, a toxic aromatic amine, is known to cause hemopoietic toxicity both in humans and animals. Aniline exposure also leads to toxic response in spleen which is characterized by splenomegaly, hyperplasia, fibrosis and the eventual formation of tumors on chronic in vivo exposure. Previously, we have shown that aniline exposure leads to iron overload, oxidative DNA damage, and increased cell proliferation, which could eventually contribute to a tumorigenic response in the spleen. Despite our demonstration that cell proliferation was associated with deregulation of G1 phase cyclins and increased expression of G1 phase cyclin-dependent kinases (CDKs), molecular mechanisms, especially the regulation of G2 phase and contribution of epigenetic mechanisms in aniline-induced splenic cellular proliferation remain largely unclear. This study therefore, mainly focused on the regulation of G2 phase in an animal model preceding a tumorigenic response. Male Sprague-Dawley rats were given aniline (0.5 mmol/kg/day) in drinking water or drinking water only (controls) for 30 days, and expression of G2 phase cyclins, CDK1, CDK inhibitors and miRNAs were measured in the spleen. Aniline treatment resulted in significant increases in cell cycle regulatory proteins, including cyclins A, B and CDK1, particularly phosphor-CDK1, and decreases in CDK inhibitors p21 and p27, which could promote the splenocytes to go through G2/M transition. Our data also showed upregulation of tumor markers Trx-1 and Ref-1 in rats treated with aniline. More importantly, we observed lower expression of miRNAs including Let-7a, miR-15b, miR24, miR-100 and miR-125, and greater expression of CDK inhibitor regulatory miRNAs such as miR-181a, miR-221 and miR-222 in the spleens of aniline-treated animals. Our findings suggest that significant increases in the expression of cyclins, CDK1 and aberrant regulation of miRNAs could lead to an accelerated G2/M transition of the splenocytes, and potentially to a tumorigenic response on chronic aniline exposure.

## Introduction

The precise causes of cancer are still not known, but the environmental factors including environmental and occupational carcinogenic chemical exposure play a potential role in the etiology of cancer [1,2]. Aniline, a widely used industrial chemical, has been implicated in splenic toxicity including splenomegaly, hyperplasia, fibrosis, and a variety of sarcomas on chronic exposure in rats [3–8]. Splenomegaly is one of the earliest characteristics of aniline-mediated splenic damage preceding fibrosis and tumorigenesis [5,6,8–10]. Previous studies in our laboratory demonstrated that aniline exposure led to increased red pulp cellularity and increases in macrophages and fibroblasts [8–14]. More importantly, our recent studies have shown iron overload and oxidative stress with consequent increase in oxidative DNA damage and cellular proliferation in the spleen of rats following aniline exposure. Such events could potentially lead to a mutagenic and/or carcinogenic response in the spleen [15–17].

Cell proliferation plays an important role in chemical-induced cell damage, especially the injury which leads to neoplasia [18–22]. A high rate of cell proliferation and disregulation of cell cycle are fundamental ingredients in the stages of chemical-induced carcinogenic activities [18–23]. Oxidative stress is known to play a vital role in the pathogenesis of a variety of human diseases including cancer [17,24–26]. Increasing evidence supports that xenobiotics-induced oxidative stress plays a role in the regulation of cell proliferation and chemical carcinogenesis [19–22]. Cell cycle, including Gap 1(G1), synthesis of DNA (S), Gap 2 (G2) and mitosis (M), is a complex and precisely controlled process, and two central groups of regulatory proteins, cyclins and cyclin-dependent kinases (CDKs), direct the progress of a cell through the cell cycle. The G2/M checkpoint prevents cells from entering mitosis and a negligent G2/M checkpoint may lead to genomic instability and cancer risk [27,28]. The key effector of the G2/M checkpoint is the CDK1 (cdc2) kinase. Activation of this kinase following association with cyclin B, a series of phosphorylation and dephosphorylation events, is essential in initiating mitosis [29]. Phosphorylation of the conserved threonine (Thr161) in the T-loop of CDK1 is required for activation of the cyclin B/CDK1 complex [27–29]. Our previous studies have shown that aniline exposure not only leads to both oxidative stress and cell proliferation in spleen, but also deregulation of G1 phase cyclins and increased expression of G1 phase CDKs [15–17,24]. However, the molecular mechanisms in aniline-mediated toxicity in the spleen, particularly the regulation and contribution of cyclins and CDKs in other phases of cell cycle and their potential contribution to cellular proliferation remain largely unclear.

MicroRNAs (miRNAs) are short non-coding RNAs containing about 22 nucleotides that play important roles in virtually all biological pathways in mammals and other multicellular organisms [30–32]. MiRNAs have been implicated in tumor/cancer development through modulating key cell cycle regulators and controlling cell proliferation [30,31,33]. To further unravel the molecular mechanisms of aniline-mediated cell proliferation, the current study focused on assessing the expression of cell cycle proteins and genes, especially G2 phase cyclins, CDK1, CDK inhibitors and miRNAs in an animal model preceding a tumorigenic response following aniline exposure.

## Materials and Methods

### Animals and treatments

Male Sprague-Dawley rats (~200 g), obtained from Harlan Sprague-Dawley (Indianapolis, IN), were maintained in a controlled environment animal room (temperature, 22°C; relative humidity, 50%; photoperiod, 12-h light/dark cycle) for 7 days prior to the treatments. The animals were randomly divided into two groups of six each. One group of animals received 0.5 mmol/kg/day aniline hydrochloride (~97%; Aldrich, Milwaukee, WI) via drinking water (pH of the solution adjusted to ~ 6.8), while the other group received water only and served as controls [7,8,10–13,15,16]. Choice of dose and duration of exposure was based on earlier studies [7,8,10,11,13,15,16]. After 30 days, the animals were euthanized under nembutal (sodium pentobarbital) anesthesia and the spleens were aseptically removed, weighed and divided into several portions for use in various analyses. Portions of the spleen were snap-frozen in liquid nitrogen and stored at -80°C for RNA isolation and protein extraction. All animal experiments were performed in accordance with the guidelines of the National Institutes of Health and were approved by the Institutional Animal Care and Use Committee at the University of Texas Medical Branch.

### Western blot analysis for protein expression

Total spleen tissue lysates used for the detection of cyclin A, cyclin B1, CDK1, p21, p27, TRX-1, Ref-1and phospho-CDK1 proteins were prepared by using the lysis buffer essentially as described by the manufacturer (Cell Signaling, Beverly, MA) [14,17,24]. Western blot analyses were performed as described earlier [13,14,17,24]. Briefly, lysates containing 50 μg of total proteins from spleen extracts were resolved by SDS-PAGE (10%) and transferred to PVDF membranes (Amersham, Arlington Heights, IL). After blocking with non-fat dry milk (5%, w/v), the membranes were incubated with antibodies specific for the above mentioned proteins (Santa Cruz Biotechnology, Santa Cruz, CA) for detecting their expression. To confirm even loading, membranes were stripped and probed with a β-actin antibody (Sigma). Blots were quantitated by densitometry and normalized using the β-actin signal to correct for differences in loading of the proteins from the control and experimental groups.

### Real-time PCR analysis for cyclin A, cyclin B1, p21 and p27 gene expression in splenocytes

Real-time RT-PCR was performed essentially as described earlier [15,17,24]. Briefly, total RNA was isolated from spleen tissues using a RiboPure kit (Ambion, Austin, TX) following the manufacturer's instructions. cDNA was prepared from total RNA by using the SuperScript first-strand synthesis kit (Invitrogen, Carlsbad, CA) as per the manufacturer's manual. Real-time PCR employing a two-step cycling protocol (denaturation and annealing/extension) was carried out using a Mastercycler Realplex (Eppendorf, Westbury, NY) and the primer pairs used in the real-time PCR process are shown in Table 1. For each cDNA sample, parallel reactions were performed in triplicate for the detection of 18S and rat cyclin A, cyclin B1, p21 and p27. The reaction samples in a final volume of 25 μl contained 2 μl cDNA templates, 2 μl primer pair, 12.5 μl iQ SYBR Green Supermix, and 8.5 μl water. Amplification conditions were identical for all reactions: 95°C for 2 min for template denaturation and hot-start before PCR cycling. A typical cycling protocol consisted of three stages, 15 s at 95°C for denaturation, 30 s at 60°C for annealing, and 30 s at 72°C for extension and an additional 20-s hold for fluorescent signal acquisition.

**Table 1:** Primer sequences of cyclins A, B1, p21 and p27 for real-time PCR analysis.

Genes	Forward	Reverse
Cyclin A	aacgatgagcacgtccctac	cagctggcctcttctgagtc
Cyclin B1	catgctggactacgacatgg	ctccgtgtgggacaggtagt
p21	gagcagtgcccgagttaagg	tggaacaggtcggacatcac
p27	ggtgccttcaattgggtctc	gcttcctcatccctggacac
18S	Universal 18S Internal Standard	

### Quantitative real-time PCR for microRNAs

Total RNA, including microRNA (miRNA) from spleen tissues was extracted by using the *mir*Vana miRNA Isolation Kit (Ambion, Austin, TX), and cDNA was synthesized from total RNA by reverse transcription (RT) using a miRCURY LNA Universal RT kit (Exiqon, Woburn, MA) in accordance with the manufacturer’s instructions. Quantitative real-time PCR was performed with the converted cDNA using miRNA LNA PCR primers and SYBR Green Master Mix (Exiqon) on an Eppendorf Mastercycler Realplex (Eppendorf). 20 μl of reaction mix consisting of 8 μl of diluted cDNA, 2 μl of primers and 10 μl of SYBR Master Mix (Exiqon) was subjected to qPCR. Initial polymerase activation/denaturation was done for 10 min at 95°C followed by 40 amplication cycles of 95°C for 10 seconds and 60°C for 1 minute. At the end of final cycle, a melting curve analysis was performed for each sample. The target sequence of each miRNA is shown in Table 2 and U6 was used as an internal control. Results were converted to relative values (log 2 ratios) between controls and aniline-treated samples.

**Table 2:** Target sequences of miRNAs for real-time PCR analysis.

Genes	Target sequences	Accession number
Let-7a	UGAGGUAGUAGGUUGUAUAGUU	MIMAT0000062
miR-15b	UAGCAGCACAUCAUGGUUUACA	MIMAT0000417
miR-24	UGGCUCAGUUCAGCAGGAACAG	MIMAT0000080
miR-100	AACCCGUAGAUCCGAACUUGUG	MIMAT0000098
miR-125	UCCCUGAGACCCUUUAACCUGUGA	MIMAT0000443
miR-181a	AACAUUCAACGCUGUCGGUGAGU	MIMAT0000256
miR-221	AGCUACAUUGUCUGCUGGGUUUC	MIMAT0000278
miR-222	AGCUACAUCUGGCUACUGGGU	MIMAT0000279
U6	Reference Gene	

### Statistical analyses

All data are expressed as means ± SD. Comparison between the groups was made by *p* value determination using Student's two-tailed *t*-test (GraphPad InStat 3 software, La Jolla, CA). A *p* value of < 0.05 was considered to be statistically significant.

## Results

### The impact of aniline exposure on cyclins A and B protein expression

**Fig 1:**
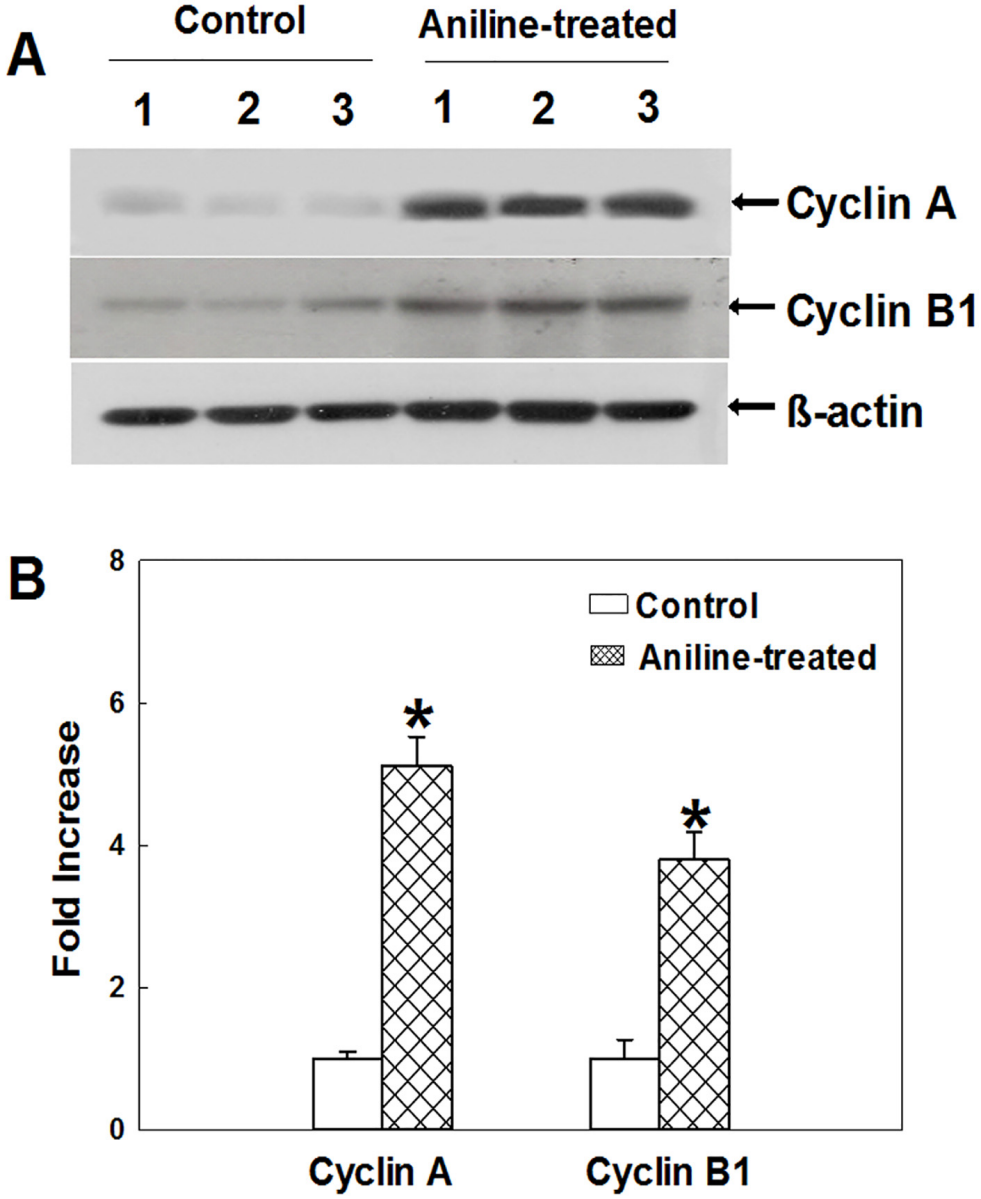
Cyclins A and B1 protein expression in rat spleens following aniline exposure (A) Western blot determination of cyclins A and B1 protein expression in control or aniline-treated rats. (B) Densitometric analysis of cyclin protein expressions. Values are mean ± SD; *p < 0.05.

Cyclins are key component of the cell cycle machinery controlling cell cycle progression [34–36]. Only after binding of cyclins, CDKs become active kinases, and those specific CDK-cyclin complexes are responsible for appropriate and ordered cell cycle transit [35,36]. CDKs are selectively activated as a result of binding of a specific cyclin. CDK1 is activated via association with cyclin A at the end of interphase to facilitate the onset of mitosis. CDK1-cyclin A complex remains into late G2 phase until replaced by CDK1-cyclin B complex and is implicated in activation and stabilization of CDK1-cyclin B which is responsible for driving cells through mitosis [34–36]. There are two different cyclin B proteins in mammalian cells. Cyclin B2 is non-essential protein during mitosis, but cyclin B1 is an essential protein that is thought to be responsible for most of action of CDK1 in the cytoplasm and nucleus [36,37]. Therefore, cyclins A and B1 were the major focus of the study and thus, analyzed. As evident from Fig 1, cyclins A and B1 protein expression in aniline-treated rat spleens showed significant increases of 412% and 280%, respectively, than the controls.

### The effect of aniline exposure on cyclins A and B mRNA expression

**Fig 2:**
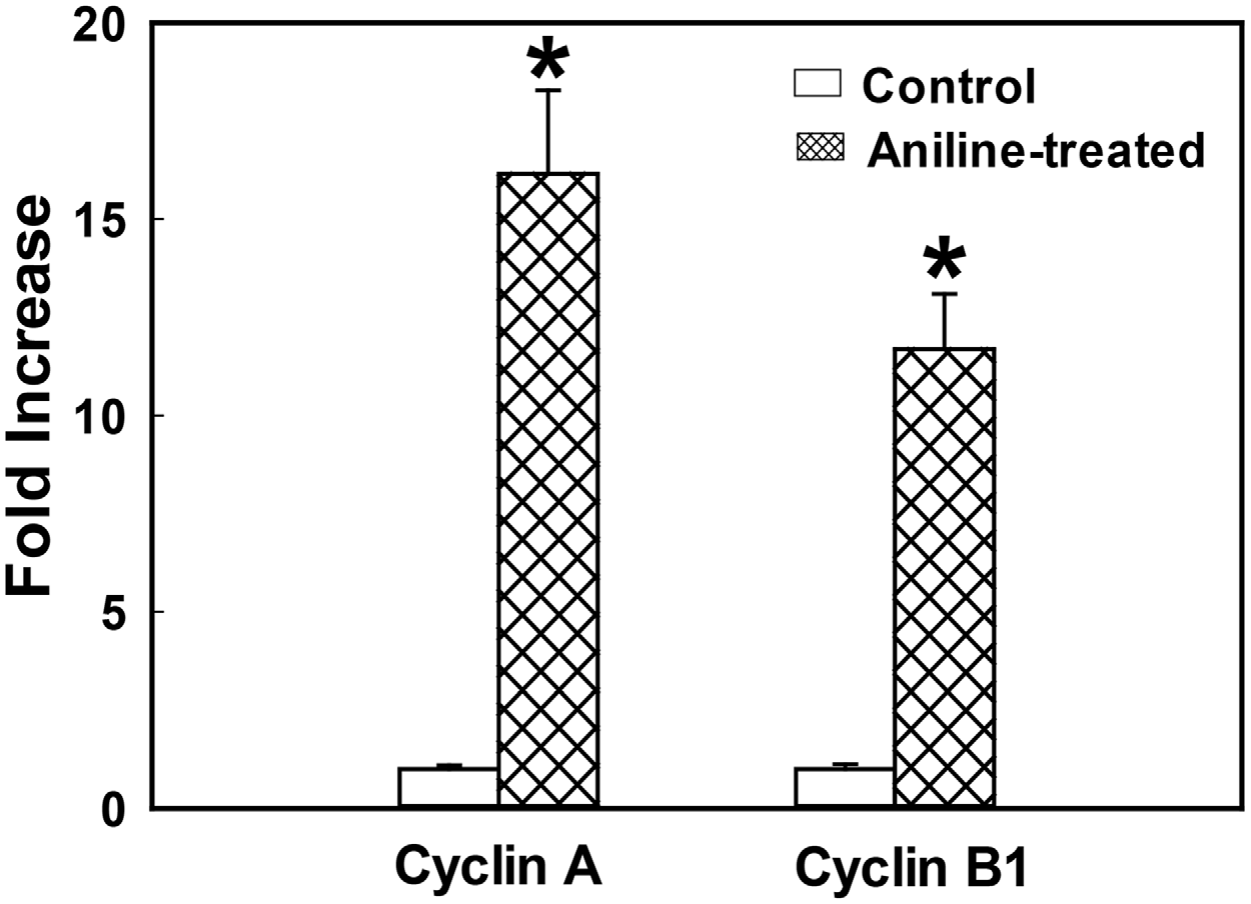
Cyclins A and B1 gene expression in rat spleens following aniline exposure. The fold change in mRNA expression (2^−ΔΔCT^) was determined by real-time PCR analysis. Values are means ± SD (*n* = 3). *p < 0.05 vs. respective controls.

As mentioned above, cyclins A and B play key role in regulating cell cycle transitions and controlling cell cycle progression via assembling with CDK1 to form cyclin-CDK complexes [34,36]. Since our Western analysis data showed that aniline exposure led to increases in protein expression of cyclins A and cyclin B1 in spleens, it was important to evaluate if aniline exposure also affected the expression of cyclins at gene levels. Therefore, the mRNA expression of cyclins A and B1 was measured by using real-time PCR and the results are presented in Fig 2. Aniline treatment led to 16 and 12 fold increases, respectively, in the mRNA expression of cyclins A and B1 in comparison to the controls, providing further support to the observed increases in protein expression.

### Aniline exposure increases the expression of CDK1

**Fig 3:**
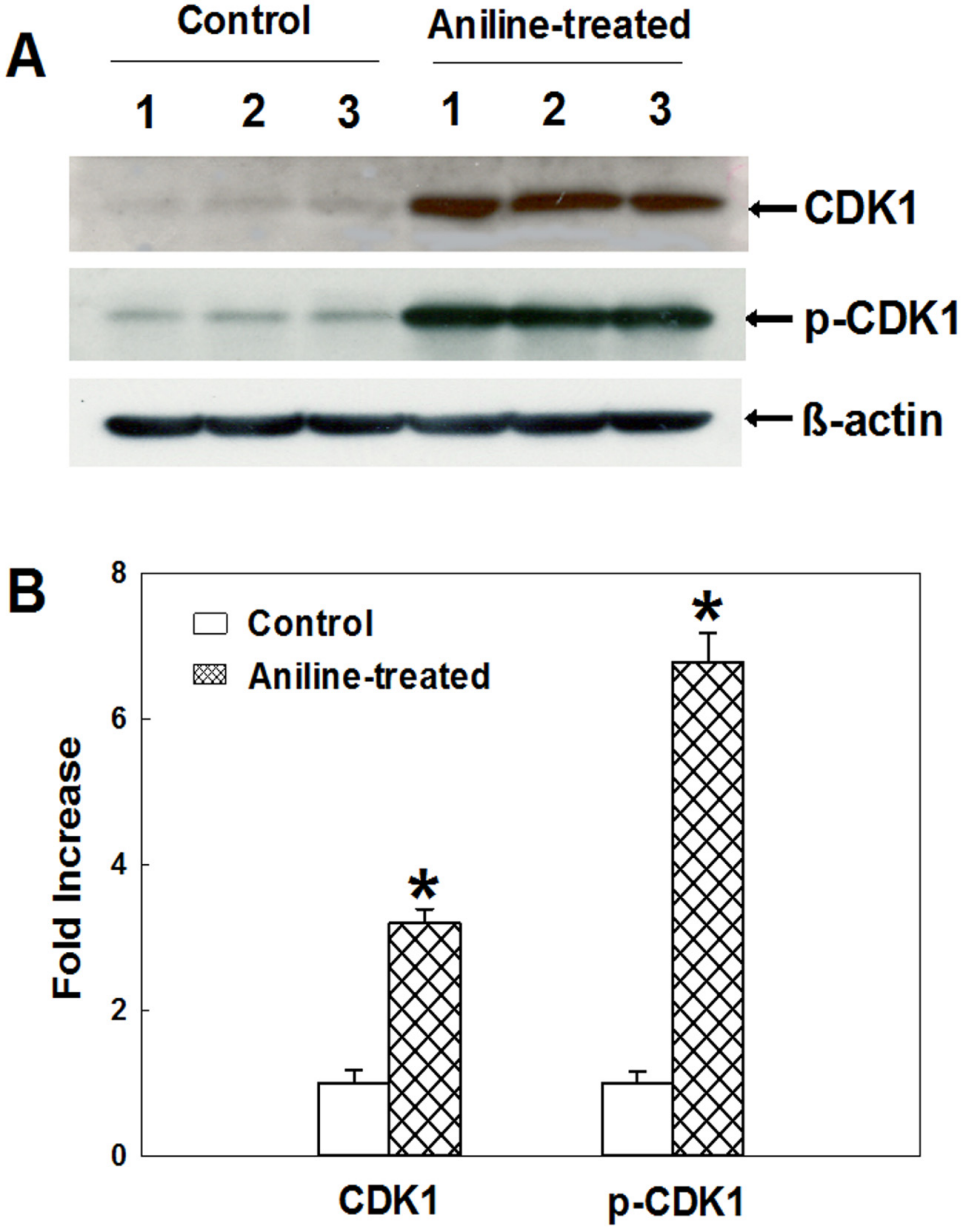
Protein expression of CDK1 and p-CDK1 in rat spleens. (A) Western blot determination of CDK1 and p-CDK1 protein expression in control and aniline-treated rats. (B) Densitometric analysis of protein expressions. Values are mean ± SD; *p < 0.05.

CDKs are central components of the cell cycle regulatory machinery and play a key role in driving cell cycle progression [27,29,34,35]. CDK1 is the master regulatory kinase of G2 checkpoint and plays pivotal role in transition from G2 to M. Phosporylation of the conserved Thr161 in the T-loop of CDK1 is required for activation of the cyclin B/CDK1 complex [27,29]. To evaluate the roles of CDK1 in regulating splenocyte proliferation, CDK1 and p-CDK1 expression was thus analyzed by Western blot and data are presented in Fig 3. The protein expression of CDK1 and p-CDK1 in aniline-treated rats showed remarkable increases of 200% and 577%, respectively, as compared to the control rats. The results further suggest that aniline treatment stimulates the expression of cell cycle regulatory proteins.

### Aniline exposure down regulates the expression of CDK inhibitors p21 and p27

**Fig 4:**
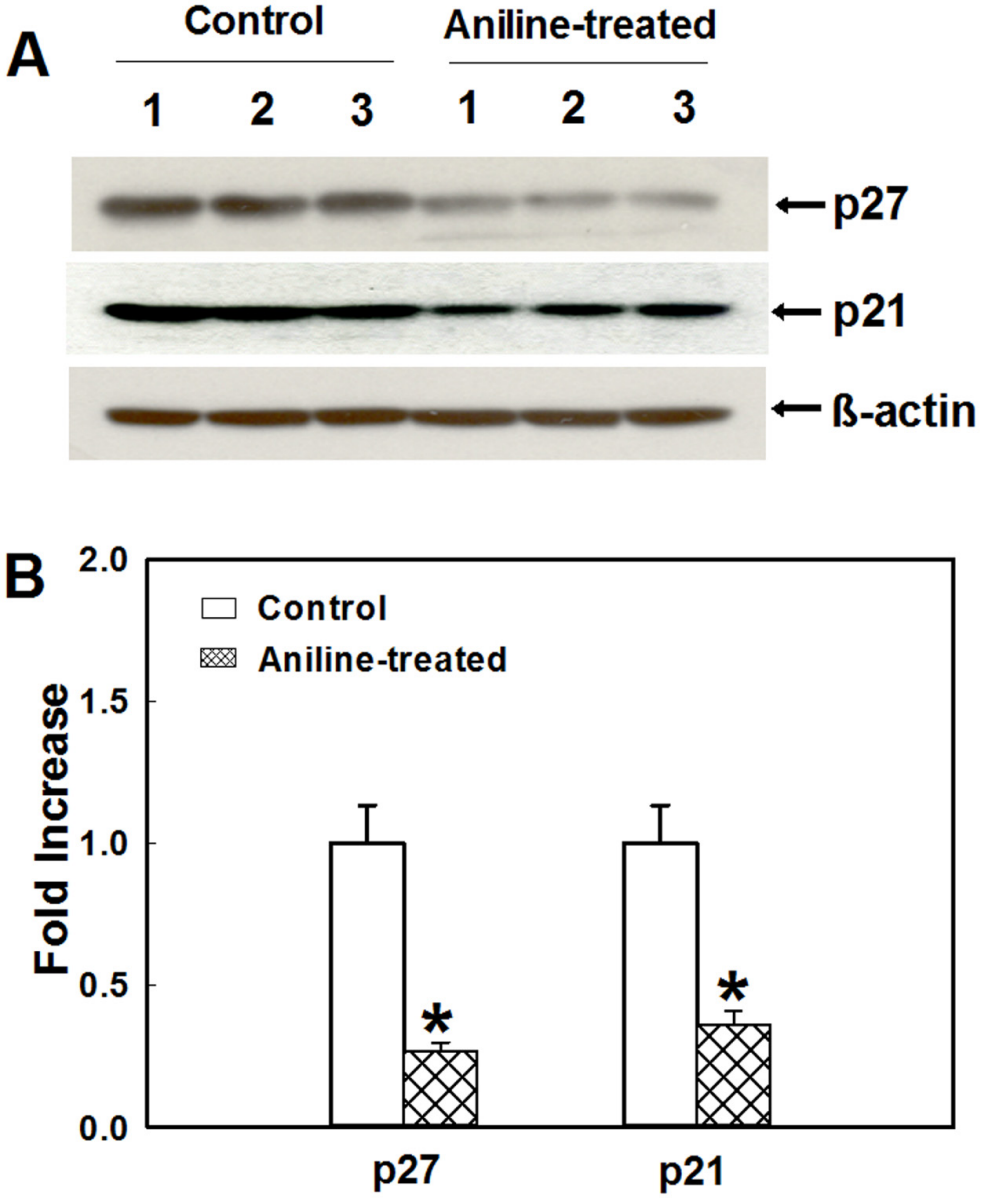
Protein expression of p27 and p21 in the rat spleens following aniline exposure. (A) Western blot detection of p27 and p21 in the spleens of control and aniline-treated rats. (B) Densitometric analysis of the protein bands. Values are mean ± SD; *p < 0.05.

**Fig 5:**
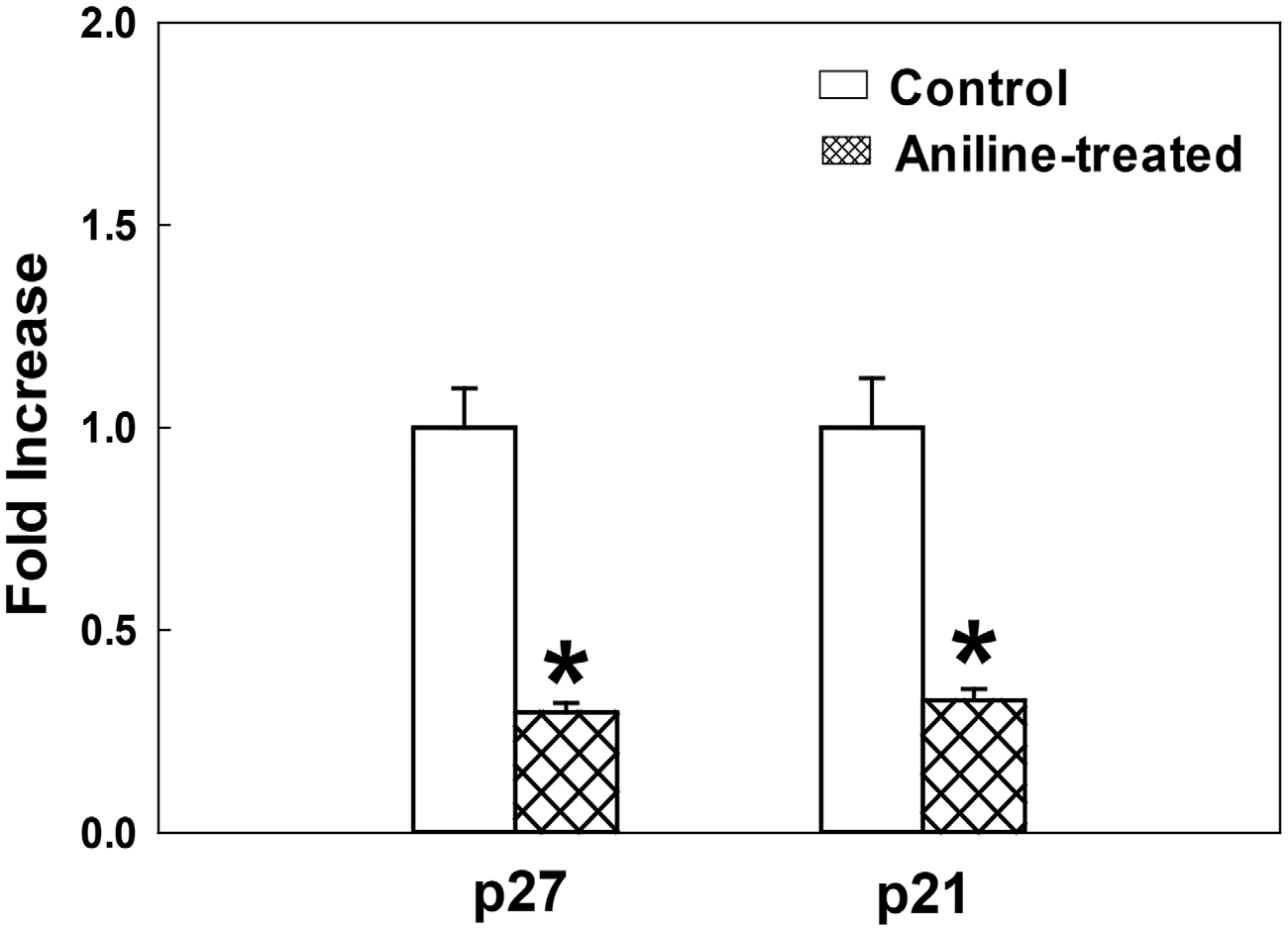
p27 and p21 gene expression in the rat spleens following aniline exposure. The fold change in mRNA expression (2^−ΔΔCT^) was determined by real-time PCR analysis. Values are means ± SD (*n* = 3). *p < 0.05 vs. respective controls.

CDK inhibitors p21 and p27 are two important cell cycle regulators. Loss of expression or function of p21 and p27 has been implicated in the genesis or progression of many human malignancies [35,38]. In addition to the role of p21 and p27 as efficient inhibitors of CDK2, CDK4 and CDK6, which also explains their ability to block cell cycle at the G1/S boundary, they have also been shown to inhibit cyclin A- and cyclin B-associated kinase activity in G2 phase [27,30,38]. Therefore, to evaluate the potential of p21 and p27 in aniline-mediated cell proliferation, the expression of p21 and p27 was also determined. As evident from Figs 4 and 5, aniline treatment resulted in significant decreases in protein expression of p21 (64%) and p27 (73%) in the spleens, whereas 67% decrease in p21 mRNA and 71% decrease in p27 mRNA expression were also observed, suggesting aniline-induced down-regulation of p21 and p27, and thus, their potential role in cell cycle progression.

### Aniline exposure promotes protein expression of Trx-1 and Ref-1

**Fig 6:**
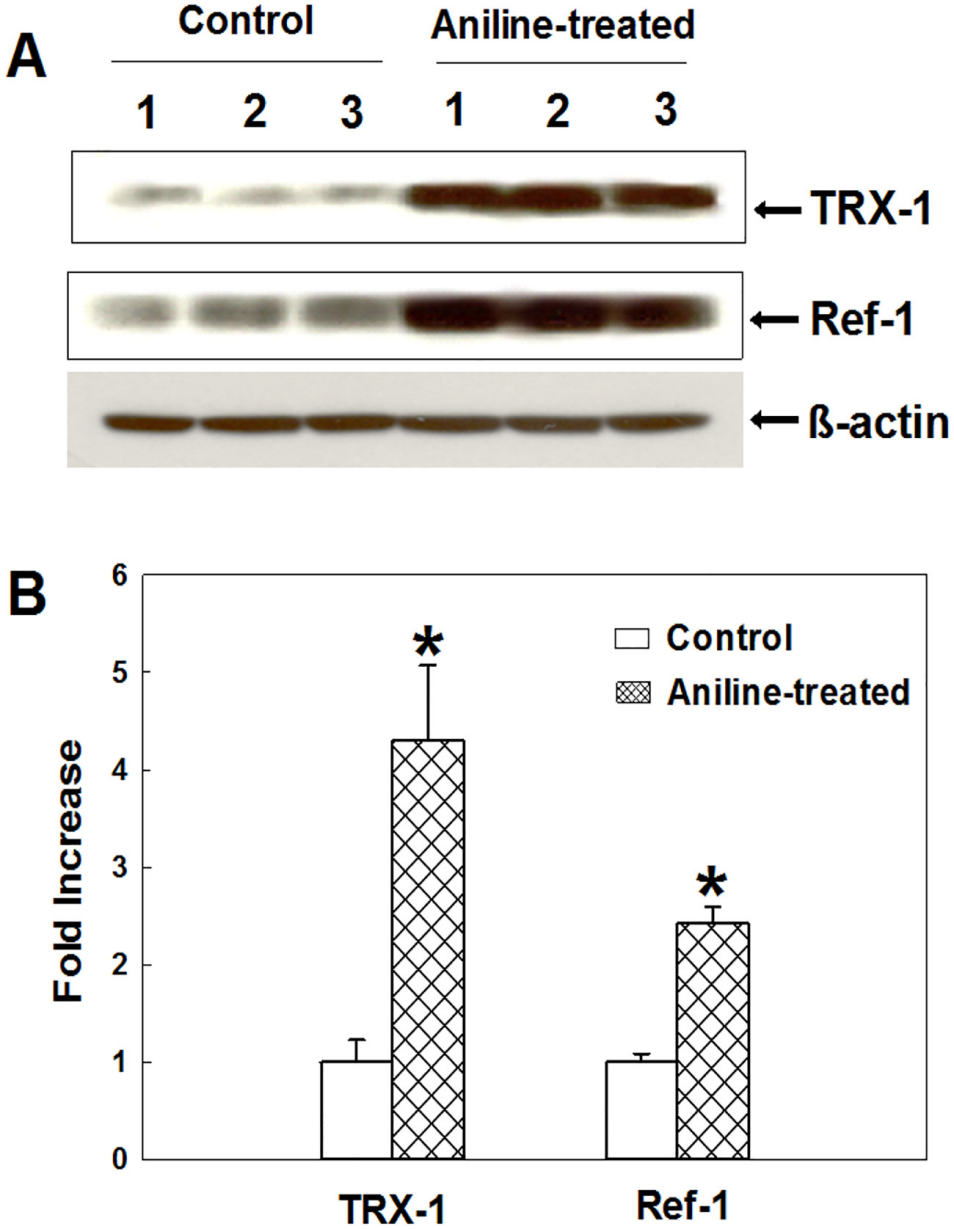
Protein expression of Trx-1 and Ref-1 in rat spleens. (A) Western blot determination of Trx-1 and Ref-1 in the control and aniline-treated rats. (B) Densitometric measurement of the protein bands. Values are mean ± SD; *p < 0.05.

Thioredoxin-1 (Trx-1) and apyrimidinic endonuclease/redoc factor-1 (APE/Ref-1) are considered hallmarks of cancer [39–41]. To determine an association between aniline exposure and potential for a carcinogenic response, the expression of Trx-1 and Ref-1 was determined. As evident from Fig 6, aniline exposure led to significant increases in Trx-1 and Ref-1 protein expression in 4.3- and 2.4-fold, respectively, in the spleens, suggesting not only an early indication but a potential for a carcinogenic response following aniline exposure.

### Impact of aniline exposure on miRNA expression

**Fig 7:**
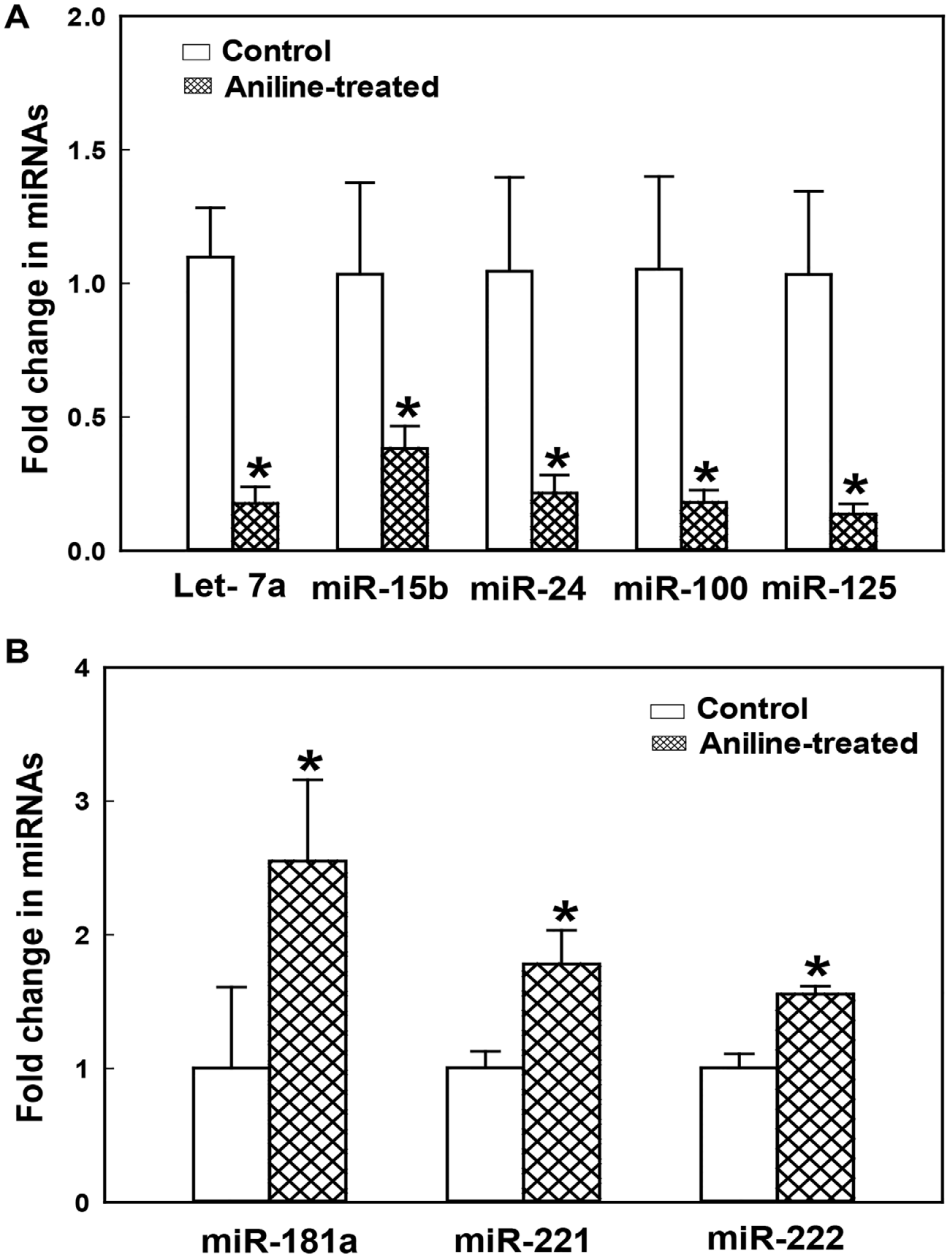
Real-time PCR analysis of miRNAs Let-7a, miR-15b, miR24, miR-100 and miR-125 (A), and miRNAs miR-181a, miR-221 and miR-222 (B) expression in rat spleens following aniline exposure. Values are means ± SD (*n* = 3). *p < 0.05 vs. respective controls.

miRNAs are functionally linked to many crucial cell-cycle control pathways. Many miRNAs are anti-proliferative and this function may be mediated by the control of different mitogenic pathways including the routes that lead to activation of cyclins and CDKs. On the other hand, several miRNAs can induce proliferation by targeting CDK inhibitors. Some miRNA may target both positive and negative regulators of the cell cycle [30–32]. To investigate whether miRNAs have a role in the cell cycle regulation of splenocytes following aniline exposure, the expression of miRNAs, including Let-7a, miR-15b, miR24, miR-100, miR-125, miR-181a, miR-221 and miR-222 which are known to mainly control G2/M phase regulators [30–32], was analyzed by using real-time PCR and the results are presented in Fig 7. Aniline exposure led to significantly decreased expression of Let-7a (decreased 82%), miR-15b (decreased 62%), miR24 (decreased 78%), miR-100 (decreased 63%), miR-125 (decreased 86%), whereas miR-181a, miR-221 and miR-222 increased by 155%, 78% and 56%, respectively, in comparison to controls (Fig 7).

## Discussion

Previous studies have shown that aniline-mediated splenotoxicity is associated with iron overload, oxidative stress and consequently increased oxidative DNA damage and cell proliferation in the rat spleens [10,12–15,17]. Recently, we not only provided a compelling evidence that aniline exposure induced greater splenocyte proliferation as analyzed by MTT assay, flow cytometry and measurement of cell proliferation markers such as proliferating cell nuclear antigen (PCNA), nuclear Ki67 protein (Ki67) and minichromosome maintenance (MCM), but also demonstrated that dysregulation of G1 phase related cyclins and CDKs, and phosphorylation of pRB which can contribute to increased cell proliferation and potentially lead to a mutagenic and/or carcinogenic response in the spleen [17]. These findings prompted us to further uncover the molecular mechanisms of aniline-mediated cell proliferation. Therefore, in this investigation, we mainly evaluated the expression of G2 phase cyclins, CDK and related regulatory genes in the spleen following aniline exposure.

Cell proliferation, as a determining factor for carcinogenicity of chemicals, is regarded to play a critical role in both the initiation and promotion stages of chemically-induced cancer [18,20–23]. Cell cycle progression plays an important role in cell proliferation, and the molecular mechanisms of cell-cycle dysregulation in aniline-mediated splenic damage remain largely unknown. To explore this, we especially focused on unraveling how aniline exposure drives the transition from G2 to M phase, a critical step in cell cycle progression. Interestingly, we not only found greatly elevated protein expression in cyclins (A and B), and CDK1, particularly p-CDK1 –the activated and required CDK1 form for activation of the cyclin B/CDK1 complexes and cell cycle progression [27,29,34], but also greater increases in the mRNA expression of cyclins A and B in the spleens of aniline-treated rats. Cell growth prior to cell division is restricted by the activity of cyclin B1/CDK1 complexes. It has been accepted that many of the G2/M regulators appear to ultimately target CDK1, the activation of which requires its association with cyclin B1 and phosphorylation at Thr161, thus controlling the G2/M phase transition [27,29,34,36]. Therefore, increased expression of cyclins and CDK1, especially the overexpression of cyclin B1 and p-CKD1 at Thr161 following aniline exposure, as observed in this study, could contribute to uncontrolled cellular proliferation in the spleen. In addition to inhibiting the activity of CDK2, CDK4 and CDK6 kinases, p21 and p27 also act as inhibitors of CDK1, the master regulatory kinase of G2 checkpoint [27,30,35,38]. Therefore, remarkably decreased expression of CDK inhibitors p21, p27 in aniline-treated spleens provides mechanistic evidence towards understanding the aniline-induced cell proliferation.

More than two centuries ago clinicians and epidemiologists first reported the induction of cancers following exposure to chemical agents [1,2]. The environmental factors, including chemicals, are implicated in ~80% of all the cancers [1,2]. Aniline, a widely used industrial chemical, is associated with splenic injury resulting in splenomegaly, hyperplasia, fibrosis, and the eventual tumor formation [3–7]. To further assess the association of aniline exposure with potential to develop splenic tumorigenesis, we determined the expression of Trx-1 and Ref-1 –two known markers of cancer [39–41], which are not only cancer markers, but also targets for cancer therapy. Expression of Trx-1 and Ref-1 increases in a wide variety of human tumors and decreases when the tumor is surgically removed. Drugs that inhibit Trx-1 and Ref-1 are in clinical development with early promising results [39–41]. Our findings in this study of increased Trx-1 and Ref-1 expression further support the potential for a carcinogenic response.

Cancer is caused by uncontrolled cell proliferation and an inappropriate survival of damaged cells, which eventually result in tumor formation. Several regulatory factors switch on or off genes that drive cell proliferation and differentiation. Aberrant expression of these genes, which are referred to as tumor-suppressor genes and oncogenes, is selective for cancer [31–33]. Increasing evidence is presented for the control of cell proliferation by miRNA and the changes of non-coding RNAs may lead to tumor formation by modulation key cell cycle regulators [31,32]. The tumor suppressor miRNAs lead to cell arrest by deregulating critical components of the cell cycle regulatory machinery. On the other hand, oncogenic miRNAs may trigger cell cycle entry and progression by targeting CDK inhibitors [30,31,33]. To investigate potential role of miRNA in aniline-induced cell proliferation and understand how aniline exposure can change the expression of cell regulators including cyclins, CDKs, CDK inhibitors, we also explored expression of miRNAs in spleen. More importantly, the miRNAs analyzed in this study not only included the miRNAs like Let-7a, miR-15b, miR24, miR-100 and miR-125 which may suppress the expression of cyclins A and B, and miRNAs such as Let-7a, miR24 and miR-125 which may regulate activity of CDK1, but also miRNAs such as miR-181a, miR-221 and miR-222 which can target CDK inhibitors [30–32]. Therefore, greater decreases in Let-7a, miR-15b, miR24, miR-100 and miR-125 expression and significant increases in miR-181a, miR-221 and miR-222 levels in the spleens following aniline treatment may be mechanistically important in generalizing that aniline exposure leads to increased cyclin A, cyclin B, CDK1, and decreased p21, p27, thus triggering the splenocytes to go through G2/M transition. Future studies using miRNA mimics and inhibitors will focus on a cause-and-effect relationship, especially to establish the role of miRNAs in the regulation of specific pathway.

Taken together, our data show that aniline exposure leads to increases in cell cycle regulatory proteins, including cyclins A, B and CDK1, particularly phosphor-CDK1, which trigger the splenocytes to go through G2/M transition. Downregulation of CDK inhibitors p21 and p27 and upregulation of tumor marker proteins Trx-1 and Ref-1 may contribute to increased cell proliferation and may be early indicators of a tumorigenic response in the spleen, respectively. More importantly, lower expression of cyclins A, B and CDK1 regulatory miRNAs and greater expression of CDK inhibitory suppress miRNAs in the spleens after aniline exposure further support that the splenocytes were promoted/primed to go through an accelerated G2/M progression and could potentially result in a tumorigenic response following chronic treatment. However, further studies are needed to better understand the precise molecular mechanisms of aniline-mediated splenotoxicity, such as the initiation of cell cycle entry and progression, and the regulatory role of epigenetic alterations including miRNA in different phases of cell cycle.
